# Topological analysis of metabolic networks integrating co-segregating transcriptomes and metabolomes in type 2 diabetic rat congenic series

**DOI:** 10.1186/s13073-016-0352-6

**Published:** 2016-09-30

**Authors:** Marc-Emmanuel Dumas, Céline Domange, Sophie Calderari, Andrea Rodríguez Martínez, Rafael Ayala, Steven P. Wilder, Nicolas Suárez-Zamorano, Stephan C. Collins, Robert H. Wallis, Quan Gu, Yulan Wang, Christophe Hue, Georg W. Otto, Karène Argoud, Vincent Navratil, Steve C. Mitchell, John C. Lindon, Elaine Holmes, Jean-Baptiste Cazier, Jeremy K. Nicholson, Dominique Gauguier

**Affiliations:** 1 Division of Computational and Systems Medicine, Department of Surgery and Cancer, Faculty of Medicine, Sir Alexander Fleming Building, Imperial College, London, SW7 2AZ UK; 2 Centre de Résonance Magnétique Nucléaire à Très Hauts Champs, 5 rue de la Doua, Villeurbanne, 69100 France; 3 Metabometrix Ltd, Prince Consort Road, London, SW7 2BP UK; 4 UMR Modélisation Systémique Appliquée aux Ruminants, INRA, AgroParisTech, Université Paris-Saclay, Paris, 75005 France; 5 Sorbonne Universities, University Pierre & Marie Curie, University Paris Descartes, Sorbonne Paris Cité, INSERM, UMR_S 1138, Cordeliers Research Centre, Paris, 75006 France; 6 The Wellcome Trust Centre for Human Genetics, University of Oxford, Roosevelt Drive, Headington, Oxford, OX3 7BN UK; 7 MRC-University of Glasgow Centre for Virus Research, Glasgow, G61 1QH UK; 8 Key Laboratory of Magnetic Resonance in Biological Systems, State Key Laboratory of Magnetic Resonance and Atomic and Molecular Physics, Wuhan Centre for Magnetic Resonance, Wuhan Institute of Physics and Mathematics, University of Chinese Academy of Sciences, Wuhan, 430071 China; 9 Centre for Computational Biology, University of Birmingham, Haworth Building, Birmingham, B15 2TT UK

**Keywords:** ^1^H NMR, Metabolomics, Transcriptomics, Genome Mapping, mQTL, eQTL, Metabolic networks

## Abstract

**Background:**

The genetic regulation of metabolic phenotypes (i.e., metabotypes) in type 2 diabetes mellitus occurs through complex organ-specific cellular mechanisms and networks contributing to impaired insulin secretion and insulin resistance. Genome-wide gene expression profiling systems can dissect the genetic contributions to metabolome and transcriptome regulations. The integrative analysis of multiple gene expression traits and metabolic phenotypes (i.e., metabotypes) together with their underlying genetic regulation remains a challenge. Here, we introduce a systems genetics approach based on the topological analysis of a combined molecular network made of genes and metabolites identified through expression and metabotype quantitative trait locus mapping (i.e., eQTL and mQTL) to prioritise biological characterisation of candidate genes and traits.

**Methods:**

We used systematic metabotyping by ^1^H NMR spectroscopy and genome-wide gene expression in white adipose tissue to map molecular phenotypes to genomic blocks associated with obesity and insulin secretion in a series of rat congenic strains derived from spontaneously diabetic Goto-Kakizaki (GK) and normoglycemic Brown-Norway (BN) rats. We implemented a network biology strategy approach to visualize the shortest paths between metabolites and genes significantly associated with each genomic block.

**Results:**

Despite strong genomic similarities (95–99 %) among congenics, each strain exhibited specific patterns of gene expression and metabotypes, reflecting the metabolic consequences of series of linked genetic polymorphisms in the congenic intervals. We subsequently used the congenic panel to map quantitative trait loci underlying specific mQTLs and genome-wide eQTLs. Variation in key metabolites like glucose, succinate, lactate, or 3-hydroxybutyrate and second messenger precursors like inositol was associated with several independent genomic intervals, indicating functional redundancy in these regions. To navigate through the complexity of these association networks we mapped candidate genes and metabolites onto metabolic pathways and implemented a shortest path strategy to highlight potential mechanistic links between metabolites and transcripts at colocalized mQTLs and eQTLs. Minimizing the shortest path length drove prioritization of biological validations by gene silencing.

**Conclusions:**

These results underline the importance of network-based integration of multilevel systems genetics datasets to improve understanding of the genetic architecture of metabotype and transcriptomic regulation and to characterize novel functional roles for genes determining tissue-specific metabolism.

**Electronic supplementary material:**

The online version of this article (doi:10.1186/s13073-016-0352-6) contains supplementary material, which is available to authorized users.

## Background

Type 2 diabetes mellitus is a prime example of a multifactorial disease, combining genetic risk factors and environmental influences, including the gut microbiome [1]. Complexity in diabetes etiology and pathogenesis relates to the existence of numerous risk genes, which often lack clear biological roles and have small effects on relevant disease traits [2–8], and the contribution of organ-specific cellular mechanisms to hyperglycemia and complications through impaired insulin secretion in pancreatic β cells and insulin resistance in central and peripheral tissues [9, 10]. Although genome-wide association studies (GWAS) have identified many diabetes risk variants [6, 7, 11–13], the underlying mechanisms remain elusive. Functional annotation of disease risk loci can progress with advances in high-density molecular phenotyping approaches, mainly transcriptomics [14–17] and metabonomics [11, 18], which inform about gene and metabolite networks for various tissues. Combining these high-throughput technologies generates complementary, and potentially convergent, multidimensional information on the function of the genome and individual genes.

Metabolic phenotyping (i.e., metabotyping) [19, 20] relates to quantitative physiological and biochemical changes to both phenotypic and genetic variation. Metabotypes provide a read-out for gene–environment interactions including microbiome influences [21, 22]. Metabotyping is particularly appropriate to define biomarkers associated with diabetes risk [9, 11, 23]. In previous studies, we and others have demonstrated genome mapping of ^1^H NMR quantitative metabotypes in mouse and rat genetic crosses and defined causal relationships between segregating genetic polymorphisms and variations in metabolite abundance [18, 24–28]. Implementation of this strategy in humans remains limited to genetic associations in blood [29–33] and urine [9, 32, 34] but will most likely progress with genetic analysis of metabolic regulation in many organs from many individuals [35, 36]. Meanwhile, rodent models of complex disorders represent useful systems to investigate the direct molecular consequences of naturally occurring genetic polymorphisms and to understand the genetic architecture of metabolic regulation in biofluids [18, 26, 37] and organs [38–40].

The integrative analysis of expression quantitative trait loci (eQTL)-associated gene networks [15, 41] and metabolomic quantitative trait loci (mQTL)-associated metabolite and candidate gene networks remains a challenge [2, 42]. Here, we present an integrative systems genetics approach designed to identify mechanistic relationships between linked alleles in genomic blocks and metabolism using ^1^H NMR-based metabotyping combined with genome-wide transcriptomic analyses. In a previous study we identified QTLs for adiposity (retroperitoneal fat pad weight) and insulin secretion on chromosome 1 [9, 43], which enabled the congenic breeding program to confirm the original associations [11, 39]. We selected a series of 12 congenic strains carrying contiguous regions of various lengths (1–177 Mb) of chromosome 1 of the spontaneously diabetic (type 2) Goto-Kakizaki (GK) rat transferred onto the genomic background of normoglycemic Brown-Norway (BN) rats for metabolic and gene expression profiling in adipose tissue. We then carried out a joint eQTL and mQTL analysis and mapped the associated genes and metabolites onto metabolic networks. Through topological analysis, we implemented a parsimonious approach (i.e., Occam’s razor) by minimizing shortest paths across the resulting networks to identify pairs of mechanistically connected eQTL-associated genes and mQTL-associated metabolites for rapid validation in cell-based assays. Our approach indicates that distinct blocks of genetic polymorphisms differentially impact the adipose tissue metabolic network, thereby prioritizing candidates for gene silencing in adipocytes, exemplified by *Galm* and *Asns*.

## Methods

### Animals

**Fig. 1:**
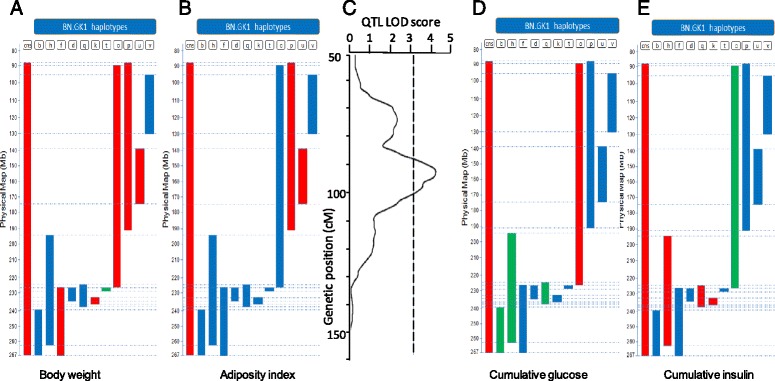
Phenotypic features of the congenic strains. Association for body weight (**a**), adiposity index (**b**), QTL for adiposity index (**c**), association for cumulative plasma glucose (**d**), and insulin (**e**) during the intravenous glucose tolerance and insulin secretion tests were measured in male rats. *Solid bars* represent the GK genomic segments of chromosome 1 of each congenic strain introgressed onto the genetic background of the BN strain. The *y-axis* shows genomic length (Mb) and boundaries of the genomic region of GK origin. The location of the adipose tissue QTL mapped to chromosome 1 in the GK × BN F2 cross [9] is reported with significance threshold shown with a *dashed line* (**c**). Details of GK chromosomal regions introgressed in each congenic strain are given in Table 1. Significantly (*P* < 0.05) increased and decreased values of the phenotypes between congenic strains and the BN control are indicated in *red* and *green*, respectively. Phenotype data are available in Additional file 1: Table S1

A colony of GK/Ox rats bred locally and derived in 1995 from the GK/Par colony was used to produce the congenic strains. BN rats were obtained from a commercial supplier (Charles River Laboratories, Margate, UK). All congenics were derived from these strains using a genetic marker-assisted breeding strategy (“speed congenics”) as previously described [11, 44] and maintained by brother–sister mating. They were specifically designed to contain GK alleles over genomic regions of various lengths (1–176 Mb) of rat chromosome 1, introgressed onto the genetic background of the BN strain (Table 1, Fig. 1). The targeted GK genomic intervals between markers D1Rat27 (90.3 Mb) and D1Got254 (264.37 Mb) cover several cardiometabolic disease (CMD) -relevant QTLs originally mapped in F2 (GKxBN) genetic crosses [9, 45, 46]. GK alleles on the X chromosome were lost early in the breeding program by two consecutive breedings of male backcross animals to BN females. All animals used in this study were systematically genotyped with markers chosen to accurately monitor retention of GK alleles across the congenic intervals and absence of GK allele contaminants from the genetic background [47, 48]. All strains were co-housed in order to avoid cage-specific microbiome selection [14, 49].

**Table 1:** Genomic details of the BN.GK congenic strains

Congenic name	Last marker BN allele	First marker GK allele	Last marker GK allele	First marker BN allele	Genomic length (Mb)
1cns	D1Got96 (88.6)	D1Rat27 (90.3)	D1Got353 (266.9)	- (Telomere)	176.6–179.3
1b	D1Rat77 (238.0)	D1Got237 (246.7)	D1Got353 (266.9)	- (Telomere)	20.2–29.9
1d	J576143 (225.8)	J337594 (227.9)	D1Got231 (231.6)	D1Got224 (233.0)	3.7–7.2
1f	J576143 (225.8)	J337594 (227.9)	D1Got353 (266.9)	- (Telomere)	39.0–42.1
1h	D1Got337 (193.5)	D1Got338 (197.0)	D1Rat84 (259.3)	D1Cebr4 (263.8)	62.34–70.3
1k	D1Got231 (231.6)	Glis3 (231.9)	XM_219778 (233.1)	Fxna (233.3)	1.2–1.7
1o	D1Got96 (90.3)	D1Got100 (92.1)	D1Wox86 (224.7)	J576143 (225.8)	132.6–137.2
1p	D1Got96 (88.6)	D1Rat27 (90.3)	D1Smu5 (189.5)	D1Got172 (191.7)	99.2–103.1
1q	D1Wox86 (224.7)	J576143 (225.8)	D1Rat75 (235.1)	D1Wox89 (236.5)	9.3–11.8
1t	J576143 (225.8)	J337594 (227.9)	D1Rat223 (228.8)	D1Rat76 (230.4)	0.9–4.6
1u	D1Wox7 (139.0)	D1Wox78 (143.8)	D1Got191 (175.4)	D1Got326 (176.6)	31.6–37.6
1v	D1Wox18 (94.6)	D1Got307 (102.5)	D1Got108 (127.4)	D1Got318 (130.1)	24.9–35.5

Name and genomic position (Mb) of the genetic markers flanking the GK congenic intervals are given, along with the minimum and maximum genomic length (Mb) of the introgressed GK genomic segment. Genomic positions were taken from the rat reference genome assembly (RGSC3.4, Ensembl release 69)

Rats were allowed free access to tap water and standard laboratory chow pellets (B&K Universal Ltd, Grimston, Aldbrough, Hull, UK) and were maintained on a 12-h light–dark cycle. All rats were identified using a microchip (identity chip, Animal Care Ltd, York, UK) linked to a database specifically designed to administer the project (husbandry, phenotype scheduling, and data storage) and store genetic information and phentoypic data [48, 50].

### Phenotype analysis

Three-month-old male congenic rats and BN controls were used for all experiments. Intravenous glucose tolerance and insulin secretion tests (IVGTTs) were performed following procedures strictly identical to those consistently applied in both F2 (GK × BN) hybrids [9, 20], which we used to map diabetes QTLs in the GK, and BN.GK congenic strains derived for several GK QTLs [11, 22, 51–54]. Briefly, rats in the post-absorptive state at the end of the post-prandial glycemic response (4.5 to 5 h fasting from 9–9:30 am when food was removed until 2 pm when the IVGTT procedures were initiated) were anesthetized by injection of 95 mg/kg body weight intraperitoneal ketamine hydrochloride (Ketalar, Parke-Davies, UK). Rats were injected with a solution of 0.8 g glucose/kg body weight via the saphenous vein. Blood samples were collected into heparinized tubes before glucose injection and 5, 10, 15, 20, and 30 min afterward. Plasma was separated by centrifugation prior to glucose assays using a diagnostic kit (ABX, Shefford, UK) on a Cobas Mira Plus automatic analyzer (ABX, Shefford, UK) and assay of immunoreactive insulin (IRI) using an ELISA kit (Mercodia, Uppsala, Sweden). Cumulative values of plasma glucose and plasma insulin during the IVGTTs were calculated to evaluate overall glucose tolerance and insulin secretion capacity in response to glucose, respectively. At 6 months, rats were killed by terminal anesthesia following an overnight fast (16–18 h). Retroperitoneal fat pads (RFPs) were collected, weighed, snap frozen in liquid nitrogen, and stored at −80 °C until preparation of tissue extracts and RNA for analysis of the metabolome and the transcriptome, respectively. The adiposity index was calculated as the ratio between RFP weight and body weight.

### Metabotyping of white adipose tissue by ^1^H NMR spectroscopy

Tissues samples (30–50 mg) were weighed into 2-mL Eppendorf tubes and were each homogenized in 1.5 ml 50 % methanol using TissueLyser (5 min at 25 Hz; QIAGEN, Germany). The mixtures were each transferred into 3-mL glass tubes and 0.7 mL chloroform was added into each sample. The mixtures were vortexed for 1 min followed by centrifugation at ~3500 g for 25 min at 10 °C. The aqueous phase was decanted and the methanol was removed under a fume cupboard before freeze drying. The lipid phase was pipetted out and chloroform was removed under a fume cupboard. Dried extracts were reconstituted using 500 μL of 0.1 M phosphate buffer solution (10 % ^2^H_2_O/H_2_O v/v, with 0.05 % sodium 3-trimethylsilyl-(2,2,3,3-^2^H4)-1-propionate for chemical shift reference at δ0.0) in 5-mm tubes for NMR acquisition. Standard ^1^H NMR spectra were measured on a Bruker spectrometer (Rheinstetten, Germany) operating at 600.22 MHz ^1^H frequency, as described previously [18, 55]. The ^1^H NMR spectra were imported into Matlab and phase- and baseline-corrected at high resolution. The region δ5.0–4.5 was removed to eliminate baseline effects of imperfect water signal pre-saturation. Each spectrum was normalized to a constant intensity sum and each variable was mean centered. Analyses were carried out using R and Matlab.

### Orthogonal partial least squares discriminant analysis

The method allows enhanced focus on strain and diet intervention while minimizing other biological/analytical variation. Sample classes were modeled using the orthogonal partial least squares (O-PLS) algorithm. This algorithm derives from the partial least squares (PLS) regression method. In linkage analysis version, the model explains the maximum separation between genotypes Y (*coded as 0, 1, 2 for GK allele numbers*) using the NMR data X. Further details on standard O-PLS implementation in metabonomics have been given previously [56, 57]. The model coefficients locate the NMR signals significantly associated with genotypic variation in a specific genomic region Y.

### RNA preparation and Illumina bead array hybridization

Total RNA was individually isolated from 100 mg of RFP (four biological replicates per strain) using the RNeasy® 96 Universal Tissue kit (Qiagen, Crawley, UK): frozen tissue samples were transferred into cooled RNeasy® 96 Universal Tissue plates and homogenized in QIAzol Lysis Reagent using a Qiagen Tissue Lyser. Following phase separation after addition of chloroform, total RNA was purified with RNeasy columns using a spin technology according to the manufacturer’s guidelines and eluted in RNase-free water. RNA concentrations were determined using a NanoDrop spectrophotometer and RNA quality, purity, and integrity were assessed using an Agilent 2100 Bioanalyser (Agilent Technologies, Waldbronn, Germany).

Samples were independently used to hybridize Gene Expression Sentrix® BeadChip RatRef-12 v1 arrays (Illumina Inc., San Diego, CA, USA) containing 22,523 oligonucleotide probes (replicated 30 times on average). They allowed interrogation of transcript levels for 21,910 genes (6274 RefSeq NM transcripts, 15,983 Refseq XM transcripts, 12 Refseq XR transcripts, 250 Unigene clusters). Double-stranded cDNA and purified biotin-labeled cRNA were synthesized from 300 ng high quality total RNA using the Illumina® TotalPrep RNA Amplification Kit (Ambion Inc., Austin, TX, USA). cRNA concentrations were determined using a NanoDrop spectrophotometer whilst cRNA quality and integrity were assessed using an Agilent 2100 Bioanalyser (Agilent Technologies, Waldbronn, Germany). Hybridizations onto the arrays were carried out using 750 ng of each (132) biotinylated cRNA in a 58 °C hybridization oven for 18 h. Following washing and staining with Streptavidin-Cy3, the BeadChip Arrays were scanned on the Illumia® BeadArray Reader (Illumina Inc., San Diego, CA, USA). Resulting data were then preliminarily analyzed using the Illumina® BeadStudio Application software before undergoing comprehensive statistical analysis. Particular attention was given to the following quality control parameters: 0 ≤ G sat ≤ 1; Green 95 percentile (GP95) for consistency between arrays (around 2000); GP5 background level in the range of the low 100 s or below.

Microarray experiments were compliant with MIAME (Minimum Information About a Microarray Experiment) and both protocol details and raw data have been deposited in ArrayExpress (http://www.ebi.ac.uk/arrayexpress/) under accession number E-MTAB-1048.

### Network representation of genome–metabolome associations

In order to explore genome–metabolome associations, a functional association network was derived from metabotype and genotype correlation coefficients using the bipartite graph Rgraphsviz package from R to represent the O-PLS correlation matrix derived from the linkage analysis between NMR variables and genotypes. A cutoff was then applied to the *P* value of the Pearson’s correlation coefficient adjusted using Benjamini and Hochberg’s multiple testing correction (*P*_*BHadj*_ < 0.05); significant correlations were set to 1 and non-significant correlations were set to 0, defining the adjacency matrix for the graph. Hence, G = (N, E) specifies a graph G with N denoting the two node sets (two types of nodes: genomic regions and metabolites) and E the edge set (link between nodes, here a correlation between metabotypes and genotypes above the cutoff, i.e., *P*_*BHadj*_ < 0.05).

### Integration of eQTL-responsive genes and mQTL-responsive metabolites on KEGG metabolic networks to identify functional candidates

Differentially regulated genes and metabolites that could be associated with GK haplotypes were mapped to KEGG pathways. Metabolic pathways were imported in R using KEGGgraph [55, 58]. An in-house python script was written to generate an adjacency table. The resulting region-specific networks were mathematically formalized as a directed multilabeled graph Gm,n = (Vm,E) composed of two types of nodes Vm, where m = (“metabolic reactions”, “metabolites”), and functional equally-weighted edges corresponding to network connectivity (i.e., metabolic reactions) between enzymes and metabolites. To identify pairs of eQTL-associated genes coding for enzymes that are metabolically connected with mQTL-associated metabolites, we computed shortest paths from the eQTL genes mapped on the KEGG network to the target mQTL metabolites using the igraph R package. Defining shortest path lengths (spl) corresponds to counting the minimal number of additional reactions required to connect a given gene and a given metabolite across the metabolic network. For example, *Galm* and β-D-glucose have a spl of 0 as β -D-glucose is the product of the reaction catalyzed by *Galm* (rn:R01602). The gene *Asns* is annotated as AsnsA and AsnsB since it involves two catalytic sites for two different reactions (rn:R00256 for AsnsB and rn:R00578 for AsnsA).

### shRNA-based inhibition of *Galm* and *Asns* expression in vitro in 3T3-L1 cells

3T3-L1 fibroblasts (ATCC, Molsheim, France) were cultured in 10 % calf serum (PAA, Velizy, France) containing DMEM high glucose (Life Technologies, Saint Aubin, France). Cells were plated at 10^5^ cells/well density until confluence and differentiated into adipocytes in 10 % fetal bovine serum (FBS; Life Technologies, Saint Aubin, France) containing DMEM high glucose, IBMX, dexamethasone (Sigma-Aldrich, Saint-Quentin, France), and insulin. Differentiated 3T3-L1 cells were maintained in 5 % FBS and DMEM high glucose.

We used pGFP-V-RS-shRNA plasmids (Origene, Rockville, MD, USA) containing short hairpin RNA (shRNA) sequences specifically designed to target *Asns* or *Galm* and a puromycin resistance gene cloned between integrative long terminal repeat sequences. The Platinum-Ecotropic Retroviral Packaging Cell Line (Cell Biolabs, San Diego, CA, USA) producing host range recombinant γ-retroviruses was used for shRNA-containing viral production. Platinum E cells were maintained in DMEM supplemented with glucose and 10 % FBS, puromycin, and blasticidin (Sigma-Aldrich, Saint-Quentin, France). Transfection was performed after adapting culture medium for 3T3-L1 cells (DMEM high glucose and 10 % calf serum without antibiotics). Fugene 6 HD® (Roche, Boulogne, France) was used according to the manufacturer’s recommendation in 3T3-L1 medium. Plasmid transfection efficiency was determined by GFP fluorescence. Supernatants were collected 48 h after transfection to transduce 3T3-L1 in the presence of polybrene (Sigma-Aldrich, Saint-Quentin, France). Puromycin selection started 24 h post-transduction.

### Differentiation and glucose transport in shRNA-transfected 3T3-L1 adipocytes

For differentiation analysis, cells were first incubated in 10 % formaldehyde (Sigma-Aldrich, Saint-Quentin, France), washed with 60 % isopropanol, and dried. A solution Oil Red O (Sigma-Aldrich, Saint-Quentin, France) was added and dishes were washed with distilled water. Quantification of coloration was performed by spectrophotometry at 490 nm with a plate reader (Perkin Elmer, Villebon, France). A separate batch of cells was used for glucose transport analysis, which was determined by incubation with a solution containing 0.5 μCi tritium labeled 2-deoxy-D-glucose. Briefly, adipocytes were cultured in DMEM high glucose without FBS for 4 h and washed with a buffer containing CaCl_2_, MgCl_2_, fatty acid free BSA (PAA, Velizy, France) in PBS (Life Technologies, Saint Aubin, France), followed by 20 min of insulin stimulation (100 nM). After 10 min of incubation with labeled 2-deoxy-D-glucose, cells were washed in ice-cold PBS and collected in a solution of NaOH for radioactivity recording and protein content quantification.

## Results

### Pathophysiological features of the congenic series

To attach specific patterns of diabetes intermediate phenotypes to each BN.GK congenic strain used in the study (Table 1) glucose tolerance, in vivo insulin secretion, body weight, and adiposity index were determined for each animal at 3 months. Rats from different strains were co-housed in the same cage to avoid cage-specific microbiome selection [14, 17, 59]. As previously observed [11], the strain carrying the largest GK haplotype (179.3 Mb in BN.GK1cns) exhibited increased body weight (270.0 ± 5.7 versus 243.4 ± 3.5) and adiposity (0.416 ± 0.028 versus 0.376 ± 0.031), glucose intolerance (CumG, 5504 ± 135 versus 5104 ± 83) and enhanced glucose-induced insulin secretion (CumI, 70.21 ± 11.50 versus 43.45 ± 3.23). To dissect the genetic basis of body weight and glucose homeostasis on chromosome 1, we validated these phenotypes in congenic substrains containing smaller GK genomic blocks introgressed into the BN normoglycemic genome (Fig. 1). Validation was achieved in nine congenic strains, which exhibited significant changes in body weight (Fig. 1a; BN.GK1f, 1k, 1o, 1p, 1t, 1u) and adiposity (Fig. 1b; BN.GK1p, 1u) when compared to BN (Additional file 1: Table S1). Rats of congenic strains BN.GK1cns, 1p and 1u showed consistent increases in both body weight and adiposity index, suggesting coordinated regulation of these phenotypes by common genetic polymorphisms in the shared GK genomic region (143.8–175.4 Mb) of rat chromosome 1 corresponding to a region of significant linkage to adiposity in the GK × BN F2 cross (Fig. 1c). Impaired glucose tolerance in BN.GK1o (Fig. 1d) may be the consequence of impaired insulin secretion (Fig. 1e), whereas improved glucose tolerance in BN.GK1h and 1q may be caused by enhanced insulin response to glucose (Fig. 1d, e). Rats of strains BN.GK1d and 1v did not show any significant change in any of these parameters when compared to BN controls (Fig. 1; Additional file 1: Table S1).

These data underline the strong phenotypic heterogeneity in these congenic strains and the complexity of the underlying genetic regulation, even though they share 95–99 % genomic homology with the BN control. Strain-specific phenotypic features can be attached to GK genomic blocks contained in each congenic line and therefore characterize the systems-wide effects of linked GK genetic polymorphisms in each contiguous region of chromosome 1.

### ^1^H NMR-based metabotyping of adipose tissue in congenic strains

To complement pathophysiological phenotypes in the congenics with molecular phenotypes, and to investigate possible relationships between physiological and metabolic variables, we carried out ^1^H NMR metabotyping of aqueous extracts of white adipose tissue from rats of the 12 BN.GK congenic strains and the BN control (Additional file 2: Figure S1). A total of 34 metabolites were detected, 31 of which could be assigned using published data [9, 19, 46] and in-house databases (Table 2). We then built orthogonal partial least squares discriminant analysis (O-PLS-DA) models to compare metabolite patterns in each congenic strain to the BN normoglycemic control (Additional file 2: Figure S2). The robustness of each O-PLS model was tested by sevenfold cross-validation and resampling under the null hypothesis as described previously [21, 48]. The range of distribution of the Q^2^_Y_ values (0.35–0.88) for each of the congenic models suggests the presence of a clear discrimination of congenic lines (Additional file 1: Table S2). We identified 19 metabolites exhibiting variations in abundance between at least one congenic strain and the BN control (Additional file 2: Figure S3; Table 2).

**Table 2:** Summary of metabolites detected in ^1^H NMR spectra from adipose tissue extracts of congenic rats and BN controls

Metabolite	^1^H chemical shift (δ, ppm) and multiplicity
Formate	8.46 (s)
Inosine^a^	8.34 (s), 8.23 (s), 6.1 (d), 4.44 (t)
Non-assigned	8.27 (s)
Non-assigned^a^	7.68 (s)
Inosine-diphosphate	6.16 (s)
Allantoin^a^	5.4 (s)
Lipids (C[H_2_]-CH_2_-CO)	5.31 (m)
Glucose^a^	5.23 (m), 3.84
Glycerophosphocholine^a^	4.32 (m), 3.23 (s)
Lactate^a^	4.11 (q), 1.33 (d)
*Myo*-inositol	4.06 (t), 3.61 (dd), 3.52 (dd)
Creatine	3.93 (s), 3.04 (s)
β-D-glucose^a^	3.91 (dd), 3.73 (d), 3.48 (m)
Glycerol^a^	3.78 (m), 3.66 (dd), 3.64 (dd), 3.56 (dd)
3-Methyl-histidine^a^	3.7 (s)
Taurine 3.42 (t)^a^	3.42 (t), 3.25 (t)
*Scyllo*-inositol^a^	3.36 (s)
Choline^a^	3.21 (s)
Non-assigned	3.13 (s), 4.04 (d)
Lipids (C = C-CH_2_-C = C)	2.76 (m)
Glutamine^a^	2.46 (m), 2.14 (m)
Succinate^a^	2.41 (s)
Glutamate^a^	2.36 (dt), 2.04 (m)
3-Hydroxybutyrate^a^	2.31 (m), 1.2 (d)
Lipids (CH_2_-CO)	2.26 (m)
*N*-acetylglutamine^a^	2.02 (s)
Acetate^a^	1.92 (s)
Lipids (C[H_2_]-CH2-CO)	1.58 (m)
Alanine	1.48 (d)
Lipids ((−CH_2_-)n)	1.27 (m)
Valine	1.04 (d), 0.99 (d)
Isoleucine^a^	1.01 (d)
Leucine	0.96 (dd)
Lipids (CH_3_)	0.89 (m)

^a^Compounds that were statistically significant (*P* < 0.05) between at least a congenic strain and BN. Patterns of significant differential regulation of these metabolites in the relevant congenic strains and contributing chromosomal regions are shown in Fig. 2

*S* singlet, *d* doublet, *t* triplet, *m* multiplet, *dd* doublet of doublet, *dt* doublet of triplet

### Definition of strain-specific metabotypes in the congenic series

**Fig. 2:**
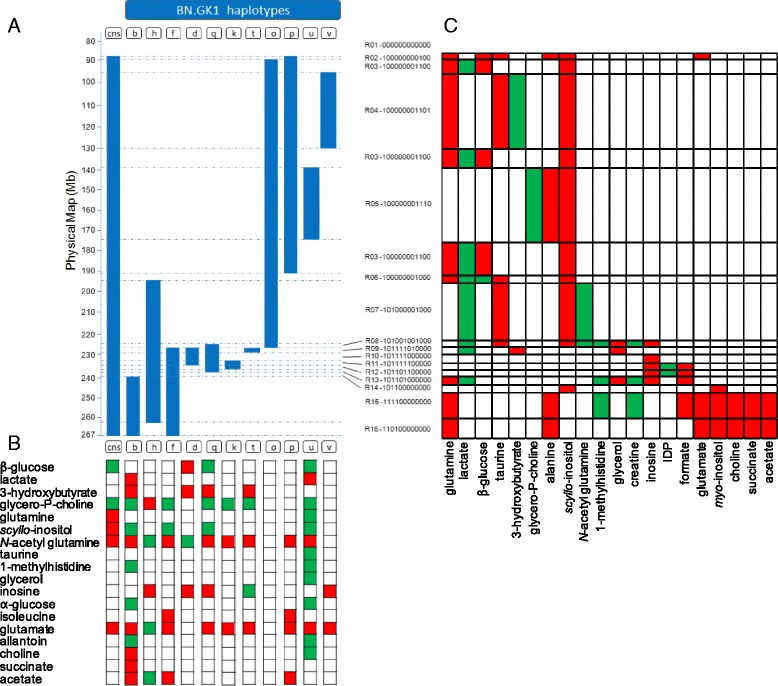
Adipose tissue metabotyping of congenic strains and haplotype-based metabotype mapping. ^1^H NMR spectra obtained at 600 MHz from adipose tissue extracts of the congenic strains and the BN controls (**a**) were used to map significant correlation networks (*P* < 0.05) between strains and metabolites in order to attach strain specific metabolite patterns (**b**) and identify chromosomal regions likely to contain GK variants responsible for variations in metabolite abundance (**c**) based on a barcode-type scoring for presence or absence of GK haplotypes. *Blue bars* represent the GK genomic segments of chromosome 1 of each congenic introgressed onto the genetic background of the BN strain. Genomic regions (R01–R16) were defined by coding the presence (1) or absence (0) of GK genotypes. *Red squares* indicate increased metabolite abundance and *green squares* increased metabolite level for each congenic strain and genomic region. Details of GK chromosomal regions introgressed in each congenic are given in Table 1. Sample numbers for each strain: BN (*n* = 5), 1cons (*n* = 4), 1b (*n* = 6), 1d (*n* = 4), 1f (*n* = 4), 1h (*n* = 4), 1k (*n* = 4), 1o (*n* = 6), 1p (*n* = 4), 1q (*n* = 5), 1t (*n* = 5), 1u (*n* = 6), 1v (*n* = 5)

To simultaneously visualize all metabotypes characterizing each congenic strain, we constructed a binary association map summarizing strain–metabolite associations (Fig. 2a; Additional file 2: Figures S2 and S3). The congenic strains BN.GK1b and 1u exhibited differential regulation of many metabolites when compared to BN. Only a few metabolites showed a strain-specific pattern of regulation, such as succinate in GK1b and taurine and glycerol in BN.GK1u. In contrast, several metabolites were differentially regulated in many congenic strains (e.g., *N*-acetylglutamine, L-glutamate, glycerophosphocholine, *scyllo*-inositol, inosine; Fig. 2a), suggesting consistent effects of common genetic polymorphisms in the shared GK genomic blocks in these strains. For example, inosine, which is consistently more abundant in BN.GK1d, 1h and 1q, may be controlled by a gene in the GK genomic block of BN.GK1d (225.8–233.0 Mb), whereas *scyllo*-inositol (δ3.36, s) was downregulated in three congenic strains (BN.GK1b, 1q, 1u) that carry distinct GK genomic blocks and is therefore controlled by different genes. Discordant trends of metabolic regulation among congenics were found for *N*-acetyl glutamine and L-glutamate, which were more abundant in adipose tissue of BN.GK1b, 1f, 1k, 1cns, 1p, 1q, 1t, and 1u than in the BN control and showed an opposite trend of regulation in BN.GK1q. These results illustrate the existence of genetically determined metabotypes and suggest functional redundancy in the regulation of individual metabolites by distinct genes.

### Identification of genetically determined metabotypes by mQTL mapping

To connect genomic information with metabolic endpoints, we took advantage of the genetic structure of the congenic panel, which is characterized by overlapping and unique GK genomic blocks across a large region of rat chromosome 1, to perform QTL analysis. The resulting association networks defined 15 independent contiguous genomic regions of GK origin across the rat chromosome 1 (Fig. 2b; Additional file 2: Figures S3 and S4). Genomic regions R15 and R16, which cover 18 Mb and 7 Mb, respectively, at the telomeric end of chromosome 1, were often associated with a similar set of metabolites (choline, L-glutamine, succinate, L-glutamate, acetate, L-alanine, *myo*-inositol; Fig. 2b). Several metabolites (L-glutamine, taurine, *scyllo-*inositol, D-glucose, L-lactate, inosine, formate) were associated with several genomic regions, suggesting the involvement of multiple independent GK variants, whereas glycerophosphocholine was specifically linked to region R5, indicating a specific effect of GK variants in this region.

### Identification of genetically determined expression traits by eQTL mapping

**Fig. 3:**
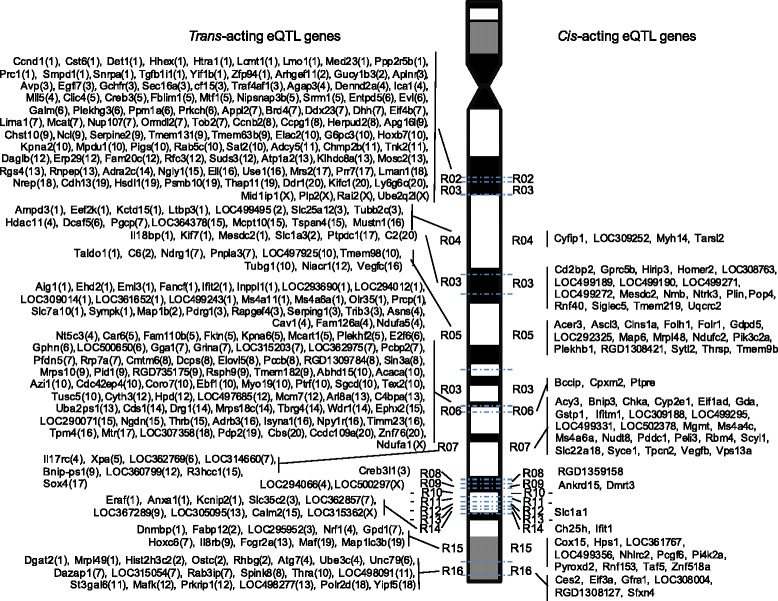
Genetic mapping of genome-wide gene expression in the adipose tissue of BN.GK congenic rats derived for chromosome 1 loci. Quantitative trait locus (QTL) mapping was applied to define regions of chromosome 1 showing evidence of statistically significant (LOD >5) linkage with changes in the transcription of genes localized in genomic regions defined by GK haplotypes (putative *cis*-acting eQTL effects) or outside the regions (*trans*-acting eQTL effects). Details of the congenic strains and congenic-defined regions are given in Table 1 and associated transcripts in Additional file 3: Table S4. The localization of genes regulated *in trans* is indicated in *parentheses*

To test the existence of functional relationships between metabolic changes mapped to congenic regions and gene expression, we next carried out genome-wide transcriptome analyses of white adipose tissue from rats of the entire congenic panel and from BN and GK controls. As previously applied to metabotypes in congenic series, we performed eQTL mapping to anchor specific gene expression patterns to each of the 15 regions of rat chromosome 1 defined by GK genomic blocks introgressed in the congenics. We found evidence of genetic linkage (LOD >5) between quantitative variation of 378 transcripts and regions of chromosome 1 (Fig. 3; Additional file 1: Table S3). Over 25 % of eQTLs correspond to genes localized within the GK genomic regions in congenics, which may underlie *cis*-mediated regulatory mechanisms. The remaining 287 eQTLs were related to transcripts localized outside the congenic region, which unambiguously underlie distant regulatory mechanisms of transcription (i.e., *trans*-acting eQTLs, as illustrated in Fig. 3).

These transcriptome data demonstrate the effect of GK variants in the congenic intervals on genome-wide gene expression and identify potential positional candidate genes that may be functionally related to changes in metabolite abundance and disease phenotypes.

### Mapping of eQTL genes and mQTL metabolites onto metabolic networks

The identification of candidate genes remains difficult because the size of the genomic blocks prevents us from applying a classic GWAS “one-SNP-one-gene-at-a-time” approach. A systems biology approach is thus required to take into account the fact that each genomic block influences multiple genes and metabotypes in a coordinated fashion, which can prove helpful to identify candidate genes relevant to diabetes and obesity. We have therefore developed a systems genetics approach stitching together genetically determined gene expression and metabolic profiles in a tissue-specific network to rank and prioritize the validation of candidate genes for adiposity and glucose homeostasis using cell-based assays.

**Fig. 4:**
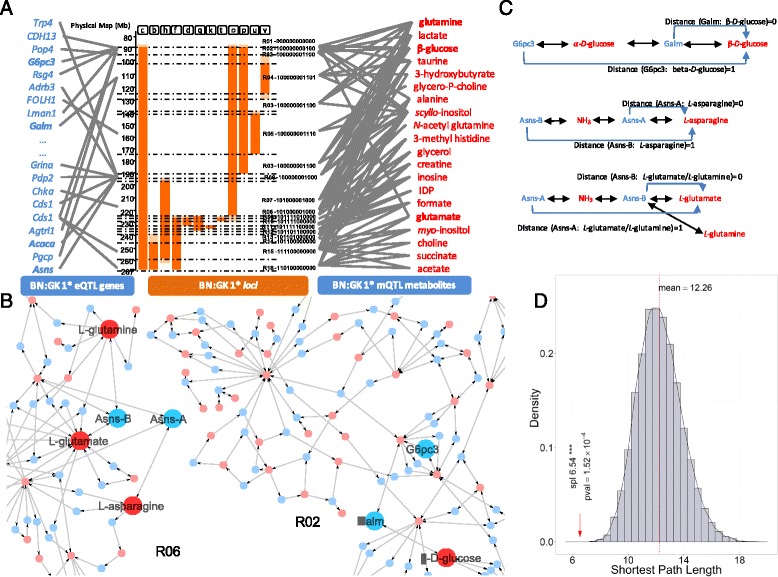
Network topological analysis of genetically regulated transcripts and metabotypes. **a** Summary of adipose transcripts and metabotypes associated with genomic regions using joint eQTL and mQTL mapping. **b** Mapping of mQTL-responsive metabolites and eQTL-responsive genes on an adipose-specific metabolic network. **c** Biologically relevant relationships between mQTL-responsive metabolites and eQTL-responsive transcripts highlighted by ranking of shortest path lengths across the metabolic network between gene–metabolite pairs (Additional file 3: Table S5). **d** Null distribution of average shortest path lengths obtained after 10,000 permutations consisting of a random selection of 20 metabolites and 73 reactions. The Asns enzyme has two catalytic sites for two reactions, which are identified as *Asns-A* and *Asns-B* in this figure

We built an association network summarizing all significant associations between genomic blocks and metabolites derived from mQTL mapping (BH adjusted *P* < 0.05) and genes derived from eQTL mapping (Fig. 4a). To systematically search for the mechanistic links between variations in metabolite abundance and gene expression, we mapped significant eQTL-responsive genes (39 genes encoding 73 reactions) and mQTL-responsive metabolites (20) onto metabolic pathways (Fig. 4b). Objective biological relationships between significant changes in gene expression and metabolite abundance were inferred following mapping of genes and metabolites to KEGG pathways and computational analysis of shortest paths between eQTL-associated genes and mQTL-associated metabolites across the metabolic pathways. Applying a shortest path length threshold of 1 (spl ≤1), we identified pairs of candidate genes and metabolites that are directly mechanistically related (i.e., the gene codes for an enzyme which directly catalyzes the reaction involving its paired metabolite; Fig. 4c; Additional file 3: Table S4). For instance, our network topology analysis showed direct connection between haplotype-associated gene *G6pc3*, encoding glucose-6-phosphatase, and glucose in chromosomal region 2. Glucose was also directly connected to *Galm*, encoding galactose mutarotase (aldose 1-epimerase). Likewise, we identified direct reactions between *Asns*, encoding asparagine synthetase (glutamine-hydrolyzing), and glutamine. To test whether the distance between the 73 reactions and 20 metabolites obtained from eQTL and mQTL mapping was significantly shorter, we permutation-tested our network under the null hypothesis: using random lists of 73 reactions and 20 metabolites for each of the 10,000 permutations and derived shortest paths. The average shortest path length of the original eQTL–mQTL directed network was 6.54 reactions, which was significantly shorter than the average shortest path length (obtained for 10,000 undirected random networks; Fig. 4d).

Through mapping eQTL-responsive genes and mQTL-responsive metabolites onto the adipose tissue metabolic network and analyzing its topology, we identified coordinated regulation of gene transcription and metabolite abundance in the adipose tissue which may account for differences in the pathophysiological phenotypes observed in these congenic strains. We therefore sought to validate by cell-based assays the relevance of the *Asns* and *Galm* genes predicted by our systems genetics approach.

### Experimental assessment of *Asns* and *Galm* function in vitro

**Fig. 5:**
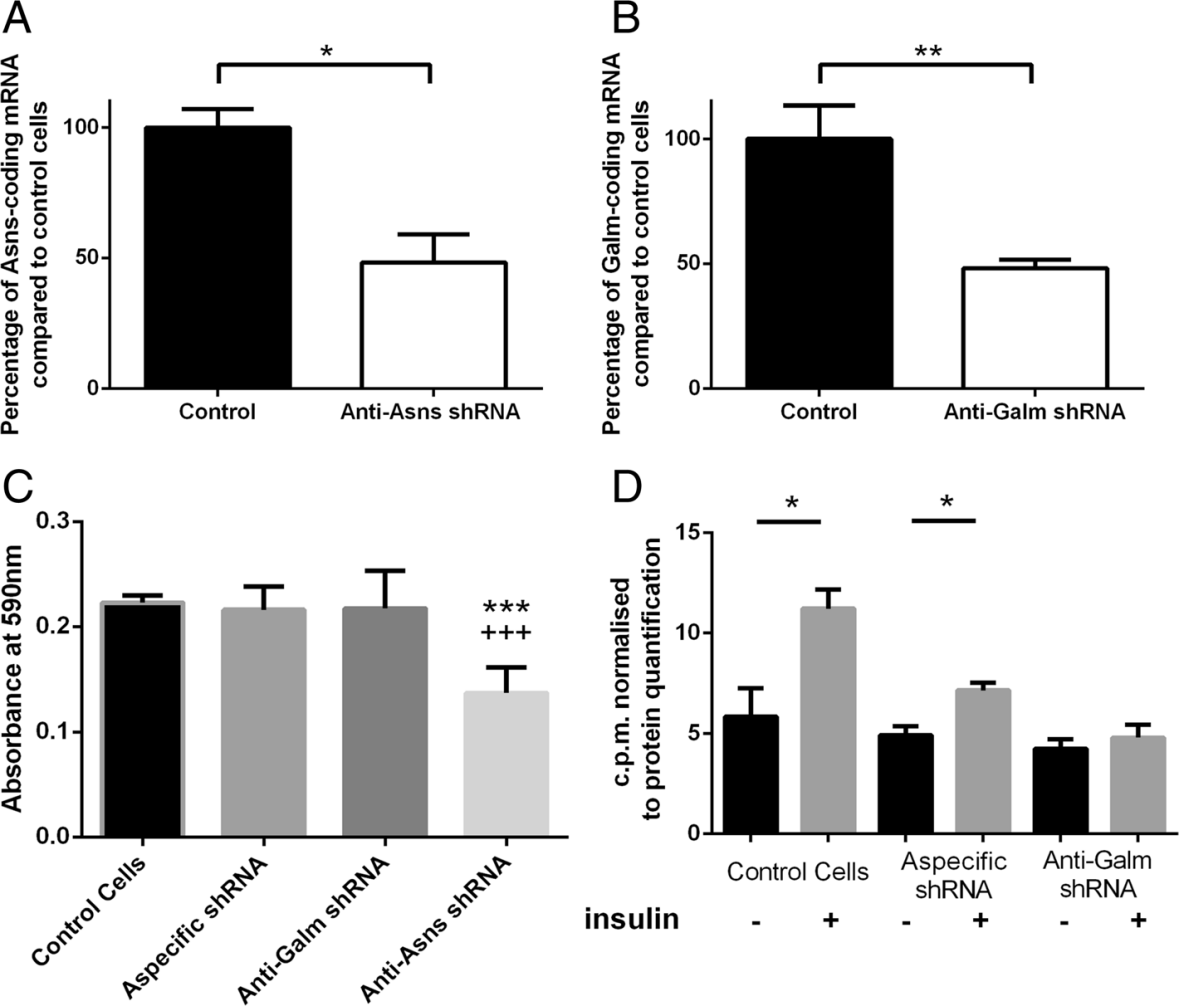
Functional assessment of in vitro shRNA-mediated inhibition of *Galm* and *Asns* expression in 3T3-L1 adipocytes. **a** *Asns* mRNA expression levels in anti-*Asns* shRNA-treated cells expressed as a percentage of control 3T3-L1 cells. **b** *Galm* mRNA expression levels in anti-*Galm* shRNA-treated cells expressed as a percentage of control 3T3-L1 cells. **c** Intracellular lipid content of differentiated 3T3-L1 cells was measured by absorbance at 590 nm after Oil Red-O staining. **d** Glucose uptake was evaluated by measurement of radiolabeled 2-deoxyglucose present in 3T3-L1 cells following insulin stimulation and normalized to protein level. Shown data represent means ± standard error of the mean. Mann–Whitney tests were performed: *P < 0.05, **P < ﻿0.01, and ***P < 0.001 significantly different to control; and ^+++^ *P* < 0.001 significantly different to *Galm*-deficient cells

To exemplify the relevance of our network integrating eQTL-responsive genes and mQTL-responsive metabolites in adipose tissue to prioritize biological validation, we selected two genes highlighted by our topological analysis: *Asns* and *Galm*. Since we had previously identified physiological QTLs mapping to chromosome 1 in the GK rat for adiposity and insulin secretion [9, 11, 14], we developed a system of shRNA-based expression knock-down for these genes in 3T3-L1 cells, which are often used to test cellular phenotypes related to diabetes (insulin-stimulated glucose uptake) and obesity (lipid accumulation through oil red-O straining) [24, 25, 27, 48]. Treatment with shRNA targeting *Asns* and *Galm* resulted in a 50 % reduction in abundance of the respective transcripts (Fig. 5a, b). The aspecific shRNA had no effect on lipid accumulation, a proxy measure of adipocyte differentiation, assessed by oil Red-O staining (Fig. 5c). Lipid accumulation was significantly hampered by *Asns* knockdown, whereas it was unchanged for *Galm* knockdown (Fig. 5c). Insulin induced a significant stimulation of glucose transport in both control cells and cells transfected with aspecific shRNA (Fig. 5d). This effect was abolished in *Galm*-deficient cells, thus demonstrating the involvement of *Galm* in the regulation of insulin signaling. The validation of the role of genes directly impacting lipid accumulation and glucose uptake in adipocytes identified in adipose tissue in a context of obesity and diabetes illustrates the efficiency of our approach based on the topology of the metabolic network.

## Discussion

Here we report the joint genome mapping of transcripts and metabolites in adipose tissue extracts using a novel network-based integration of several -omics dimensions (genome, transcriptome, and metabolome) to prioritize mechanistic investigations in adipocyte biology in a context of metabolic syndrome.

Our approach can be summarized as follows. First, we used a rat congenic panel to study glucose homeostasis and adiposity whilst limiting epistatic interactions. Second, we profiled the adipose tissue metabolome and transcriptome to identify loci regulating metabolism through differential expression and complex eQTL and mQTL patterns. Third, to tackle the complexity of these genetically determined co-regulation patterns between transcripts and metabolites, we mapped underlying eQTL-responsive genes and mQTL-responsive metabolites onto metabolic networks and minimized the search space through topological analysis within those networks, which highlighted mechanistically relevant pairs of candidate genes and metabolites. Fourth, using shortest path lengths across the metabolic network, we prioritized genes for mechanistic investigation by gene silencing and uncovered novel roles for *Asns* and *Galm* in adipocyte biology, thus validating the pertinence of our network topology analysis.

**Fig. 6:**
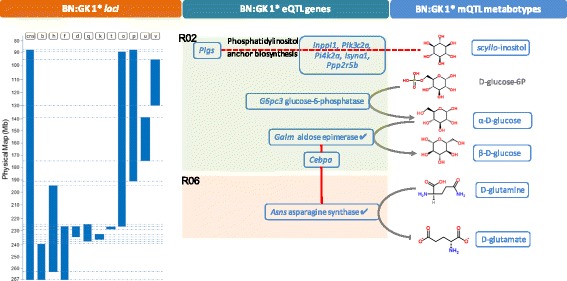
Illustration of network-based mapping of eQTL and mQTL signals. Synthetic functional map illustrating biological connections between genomic regions *R02* and *R06* of chromosome 1 and differential regulation of transcripts and metabolites

This systems genetics approach provides insights into possible coordinated mechanisms impacted by distinct series of blocks of genetic polymorphisms in well-defined genomic regions in congenic strains, as well as strain-specific phenotypic patterns. The overall interconnectedness of the association patterns between genomic blocks and metabolites illustrates the complex genetic architecture of metabolic regulation (Fig. 6). In particular, we identified coordinated changes in the regulation of metabolite abundance (D-glucose, L-glutamine) underpinned by *trans*-mediated expression of two genes (*Galm* and *Asns*) (Fig. 6) and uncovered a novel role for these genes in adipocyte function, which may have repercussions on pathophysiological phenotypes.

Our experimental approach in congenic strains allowed the dissection of the biological consequences of overlapping series of contiguous, localized GK genetic polymorphisms, thus limiting gene × gene interactions (epistatic effects) to interactions between homozygous GK variants present in the same genomic blocks. Chromosome substitution strains also demonstrated the impact of epistatic interactions on the detection and significance of genetic associations [9, 29]. We show that, even in the simplified context of congenic series, the regulation of adipose tissue metabolism involves different combinations of metabolites (i.e., specific metabotypes), thus providing experimental evidence for the strong capacity of organ metabolism to adapt to subtle genetic changes.

Such complexity of the metabolic regulation in the congenic panel was illustrated by strain-specific changes in metabolite abundance in adipose tissue, which may account for phenotypes discriminating GK and BN rats, including primarily glucose tolerance and adiposity [9]. Genome mapping of these effects indicate that GK genetic polymorphisms in several regions of rat chromosome 1 independently affect phosphatidylinositol signaling and glucose sensing and metabolism, suggesting functional redundancy of genes to ensure maintenance of essential phenotypes [35]. Prime examples are significant associations of GK haplotypes with *myo*-inositol (region 15) and *scyllo*-inositol (regions 2 and 6), which are stereoisomers of inositol, as well as with glucose and L-glutamine (regions 2 and 6) (Fig. 6). Inositol derivatives regulate insulin signaling in humans [37] and their conversion is reduced in insulin-sensitive tissues in the GK rat [38]. Chronic *myo*-inositol treatment results in improved glucose homeostasis and decreased adipocyte volume [41]. Glutamine is a TCA cycle-replenishing substrate and its analogues regulate insulin sensitivity in cultured adipocytes by preventing the desensitization of the glucose transport system [42].

Transcriptome data in the congenic strains provided possible explanations for changes in metabolite regulation, when the genetic control of metabolites and transcripts encoding biologically relevant proteins co-localizes in the same genomic region. We were able to map to the same regions of chromosome 1 the genetic control of the abundance of inositol compounds and transcripts functionally relevant to inositol metabolism, including phosphatidylinositol glycan anchor biosynthesis class S (*Pigs*; LOD = 7.42), inositol polyphosphate phosphatase-like 1 (*Inppl1*; LOD = 8.04), phosphoinositide-3-kinase, catalytic subunit type 2 alpha (*Pik3c2a*; LOD = 5.26), phosphatidylinositol 4-kinase type 2 alpha (*Pi4k2a*; LOD = 9.87), and inositol-3-phosphate synthase 1 (*Isyna1*; LOD = 5.73), which plays a critical role in *myo*-inositol biosynthesis. Furthermore, association with the transcript encoding protein phosphatase 2, regulatory subunit B' beta (*Ppp2r5b*; LOD = 6.34), regulated by *Pik3c2a*, supports the proposed role of defective regulation of serine/threonine protein phosphatases on insulin resistance in GK adipocytes [43].

Integrative analysis of multi-level -omic datasets in QTL mapping contexts remains a challenge [39], which has been best addressed in segregating populations in yeast [44] and mice [45] through the application of network biology tools. Through search space minimization within metabolic networks, we designed our metabolic network topology analysis to highlight direct coordinated functional relationships between genetically determined transcripts and metabolites and to prioritize them for experimental validation. This approach is particularly relevant compared to recent advances in the field. For instance, MetaboNetworks [47] computes the minimal network interconnecting a metabolite list but ignores gene lists. IMPaLA [49] integrates gene and metabolite lists to perform over-enrichment analyses whilst Ambient [50] agnostically identifies modules from metabolite and gene lists in the absence of arbitrarily defined pathway boundaries. These approaches can be applied to eQTL-associated genes and mQTL-associated metabolites but do not specifically rely on minimization of the search space within the network.

Through minimization of shortest path lengths between candidate eQTL genes and associated mQTL metabolites, we successfully prioritized and validated the mechanistic relevance of pairs of transcripts and metabolites, such as L-glutamine and asparagine synthetase (glutamine-hydrolyzing) (*Asns*) in region 6 of chromosome 1 corresponding to the QTL linked to adiposity in the GK × BN F2 cross [9] (Fig. 1c) or D-glucose and galactose mutarotase (aldose 1-epimerase; *Galm*) in region 2. Loci in these regions accounted for *trans*-mediated genetic control of transcripts for *Asns* (LOD = 5.70) and *Galm* (LOD = 5.34). ASNS converts glutamate and asparagine into glutamine and aspartate and GALM catalyses the interconversion of the two glucose anomers. We were able to demonstrate the roles of these genes in vitro in 3T3-L1 cells in cellular differentiation (*Asns*) and glucose uptake (*Galm*), providing new insights into their function in (pre)adipocyte physiology.

Results from functional genomic analyses in BN.GK congenics illustrate the molecular consequences of naturally occurring DNA polymorphisms originally selected in the GK strain for their role in the regulation of glucose homeostasis [51]. The >175-Mb GK genomic region in the main congenic strain (1cns) contains over 362,300 DNA variants when compared to BN, including 264 non-synonymous coding variants and exonic deletions [55], which were dissected out in shorter haplotypes (down to 1 Mb) in congenic sub-strains. A report of polymorphisms in *Inppl1* in the GK affecting insulin sensitivity [56] is consistent with our finding of disrupted regulation of metabolites involved in phosphatidylinositol signaling in adipose tissue in congenics. Furthermore, analysis of genome sequence data in 27 inbred rat strains showed that the promoter region of *Galm* contains a series of DNA variants unique to the GK [55], suggesting that they could be etiologically relevant to glucose intolerance and adiposity in the GK strain.

## Conclusions

We developed a novel network-based systems genetics [59] framework for joint mQTL and eQTL analyses of metabolic and gene expression profiles in adipose tissue of a series of rat congenic strains to dissect metabolic regulations and identified underlying physical and functional links between significant genes and metabolites, best exemplified by the novel biological roles we describe for *Galm* and *Asns* in adipocytes. Our metabolic network topology analysis approach integrates haplotype-specific co-regulated metabolites and gene transcripts, thus providing crucial information for functional annotation of genomes and for deciphering disease-associated molecular mechanisms.

## Additional files

Additional file 1: Supplementary Tables 1-3. (PDF 109 kb) Additional file 2: Supplementary Figures S1–S5. (PDF 2442 kb) Additional file 3: Supplementary Tables 4-6. (XLSX 28452 kb)
