# Supplementary material for: Topological analysis of metabolic networks integrating co-segregating transcriptomes and metabolomes in type 2 diabetic rat congenic series
Source: Genome Med. 2016 Sep 30;8:101. doi: 10.1186/s13073-016-0352-6 (PMC5045612; doi:10.1186/s13073-016-0352-6)
Supplement: Additional file 1: — Supplementary Tables 1-3. (PDF 109 kb) [file 13073_2016_352_MOESM1_ESM.pdf]

Table S1. Phenotypes in congenic and BN and GK progenitor strains. Values are means ± SE. Number of observations is reported in parentheses. ***P<0.001; **P<0.01; *P<0.05, significantly different to BN controls. Details of GK genomic regions in the congenics are given in Table 1.

strain	Body weight (g)	Adiposity	CumG (mg/dL)	CumI (µg/L)
BN	243.4 ± 3.5 (62)	0.376 ± 0.031 (40)	5104 ± 83 (39)	43.45 ± 3.23 (29)
GK	284.9 ± 5.4 (16)***	1.246 ± 0.087 (25)***	12181 ± 849 (8)***	76.05 ± 6.76 (18)***
BN.GK1b	243.4 ± 3.3 (44)	0.342 ± 0.022 (31)	4695 ± 59 (20)***	50.15 ± 4.51 (16)
BN.GK1cns	270.0 ± 5.7 (21)***	0.416 ± 0.028 (29)*	5504 ± 135 (14)***	70.21 ± 11.50 (14)*
BN.GK1f	253.5 ± 2.3 (36)*	0.375 ± 0.026 (30)	5048 ± 154 (7)	48.99 ± 3.06 (15)
BN.GK1d	249.0 ± 7.5 (20)	0.327 ± 0.025 (10)	4934 ± 129 (6)	40.07 ± 3.82 (12)
BN.GK1h	253.4 ± 8.1 (7)	0.457 ± 0.059 (11)	4849 ± 78 (6)*	67.82 ± 7.17 (10)**
BN.GK1k	258.0 ± 5.2 (22)*	0.330 ± 0.021 (15)	5074 ± 89 (11)	57.52 ± 5.18 (8)*
BN.GK1o	264.3 ± 3.9 (20)***	0.419 ± 0.032 (22)	5356 ± 65 (11)*	32.01 ± 2.85 (14)*
BN.GK1p	265.6 ± 9.5 (16)*	0.596 ± 0.044 (6)*	4977 ± 156 (7)	40.54 ± 5.01 (7)
BN.GK1q	251.6 ± 5.3 (17)	0.357 ± 0.042 (18)	4520 ± 128 (9)**	69.24 ± 6.87 (10)**
BN.GK1t	216.5 ± 5.7 (22)***	0.442 ± 0.044 (12)	4883 ± 205 (8)	47.78 ± 3.42 (11)
BN.GK1u	260.3 ± 3.7 (19)**	0.452 ± 0.038 (13)*	5018 ± 87 (11)	57.30 ± 9.80 (6)
BN.GK1v	231.6 ± 5.7 (19)	0.325 ± 0.040 (6)	5105 ± 88 (10)	50.65 ± 3.85 (11)

Table S2. Parameter values for cross-validations performed to assess the validity of the O-PLS models designed to compare metabolomic data in each BN.GK congenic strain against BN controls.

O-PLS model	R^2X	Q^2Y
1b/BN	0.542	0.737
1f/BN	0.683	0.718
1q/BN	0.753	0.879
1p/BN	0.717	0.631
1u/BN	0.870	0.735
1k/BN	0.647	0.358
1t/BN	0.758	0.592
1v/BN	0.935	0.625
1cons/BN	0.815	0.887
1d/BN	0.843	0.468
1h/BN	0.688	0.351
1o/BN	0.616	0.489

Table S3. Parameter values for cross-validations performed to assess the validity of the O-PLS models designed to compare metabolomic data attached to each chromosomal region.

O-PLS model	R^2	Q^2Y
R2/Others	0.653	0.474
R3/Others	0.650	0.242
R4/Others	0.654	0.256
R5/Others	0.540	0.136
R6/Others	0.653	0.464
R7/Others	0.653	0.477
R8/Others	0.648	0.300
R9/Others	0.579	0.005
R10/Others	0.540	-0.091
R11/Others	0.513	-0.005
R12/Others	0.633	0.027
R13/Others	0.635	0.091
R14/Others	0.645	0.060
R15/Others	0.564	0.207
R16/Others	0.568	0.257
