# Supplementary material for: Topological analysis of metabolic networks integrating co-segregating transcriptomes and metabolomes in type 2 diabetic rat congenic series
Source: Genome Med. 2016 Sep 30;8:101. doi: 10.1186/s13073-016-0352-6 (PMC5045612; doi:10.1186/s13073-016-0352-6)

Figure S1. ^{1}H 600 MHz metabolomic spectra of adipose tissue extracts from the twelve BN.GK congenic strains and the BN control.

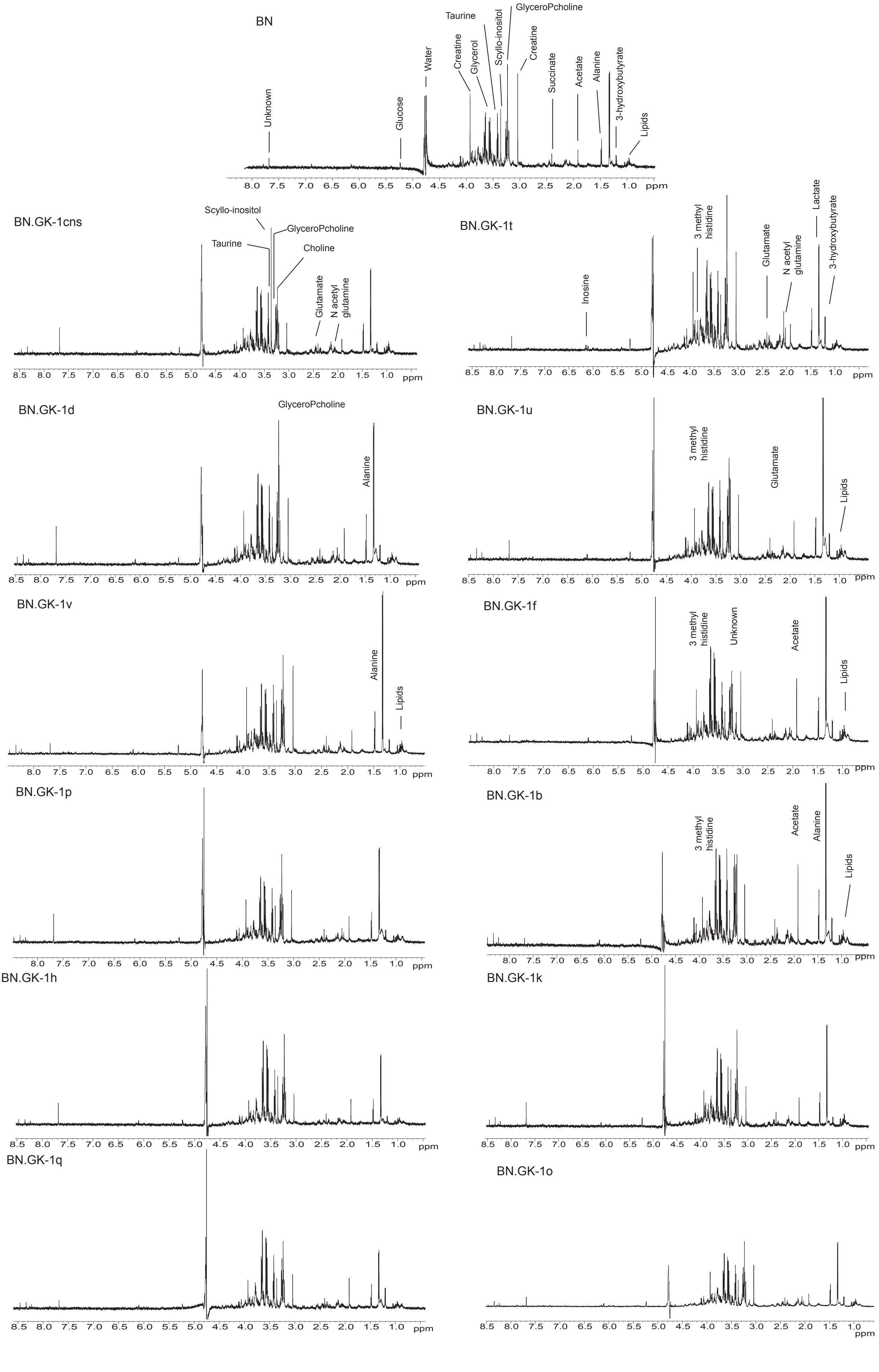

Figure S2. Score plot from O-PLS model of each BN.GK congenic strain against the BN control and corresponding cross-validation with 200 permutations

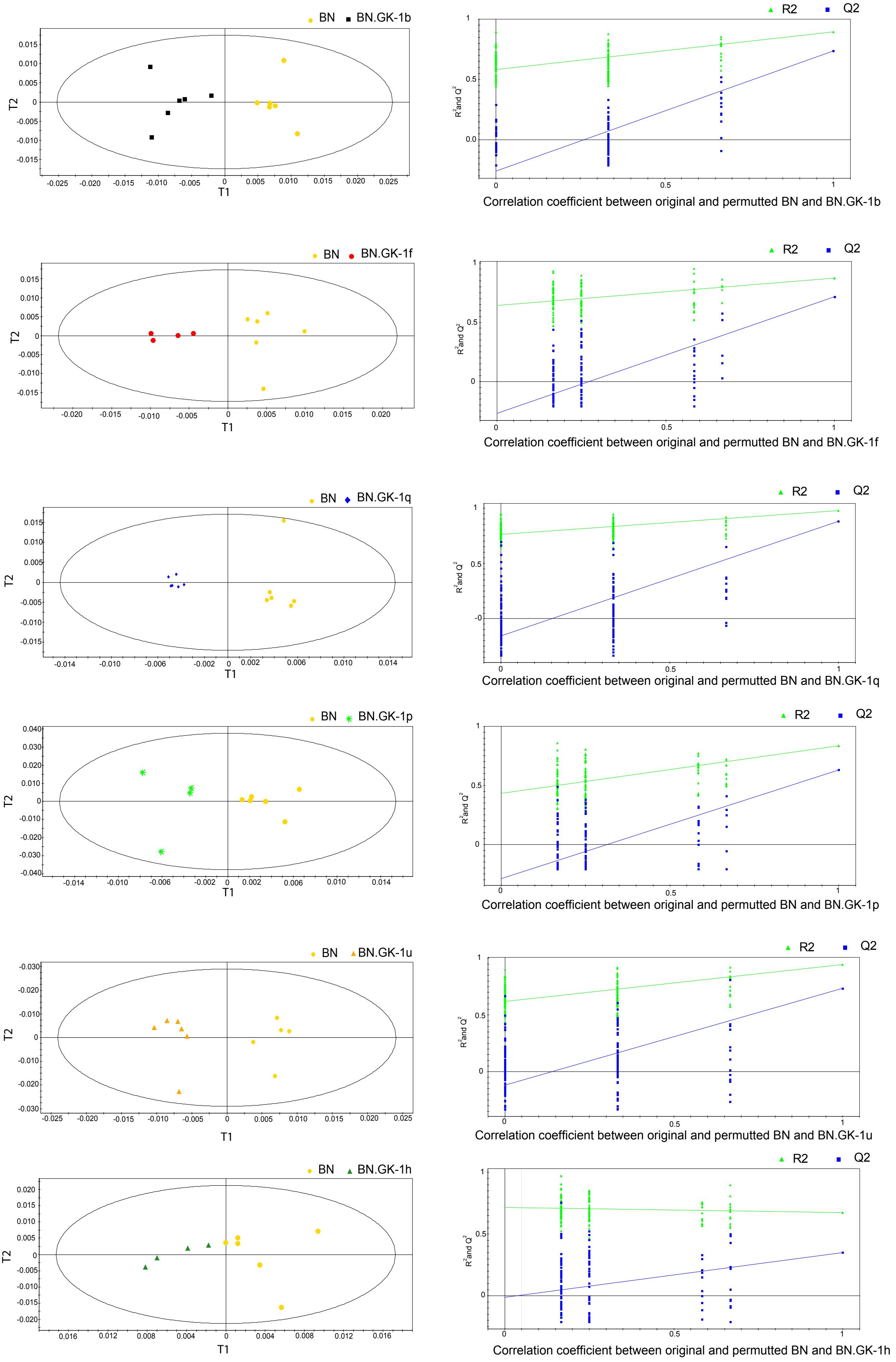

Fig S2 (cntd)

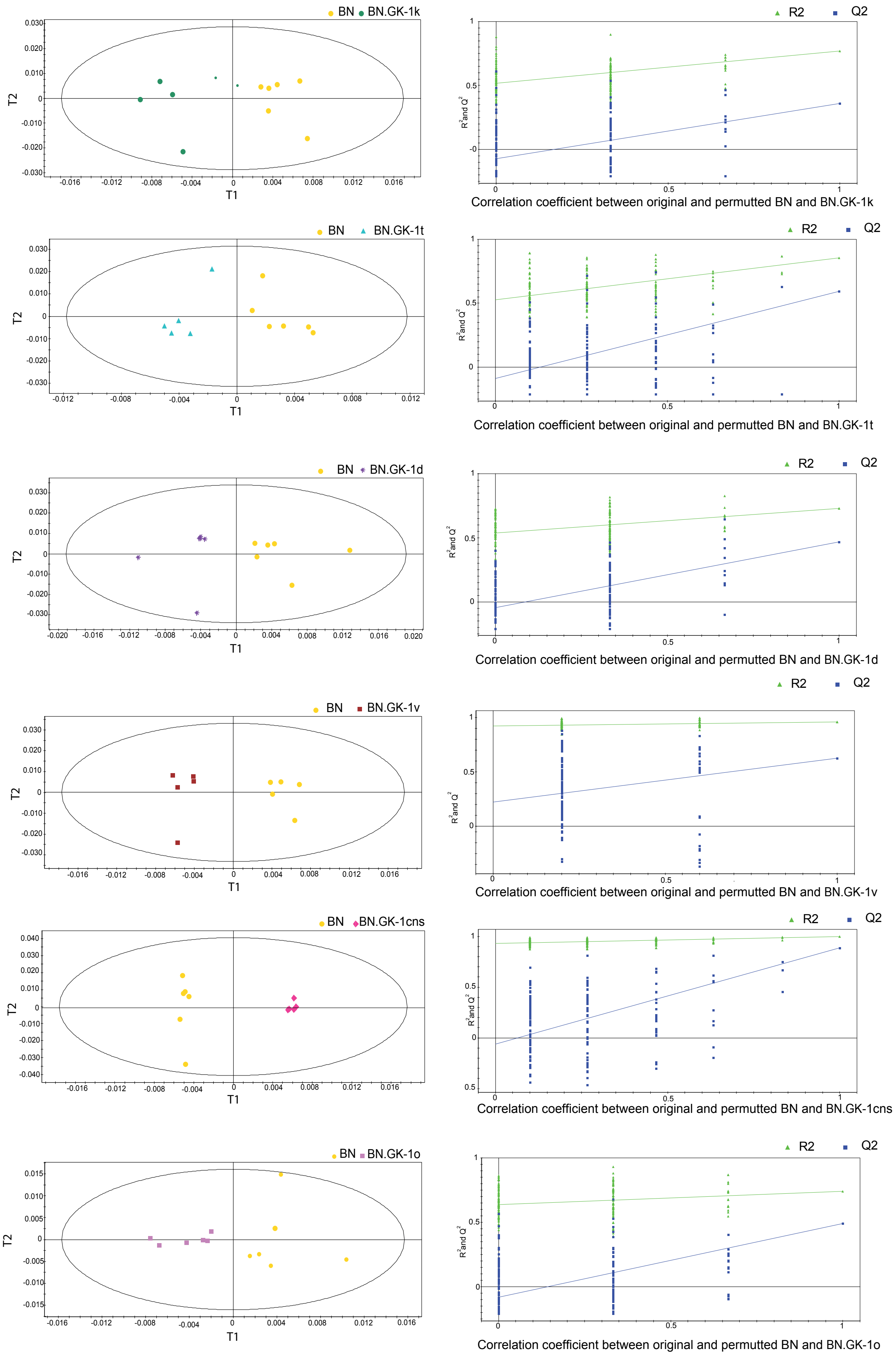
BN BN.GK-1k
T2
T1
R2 Q2
R²and Q²
Correlation coefficient between original and permutted BN and BN.GK-1k
BN BN.GK-1t
T2
T1
R2 Q2
R²and Q²
Correlation coefficient between original and permutted BN and BN.GK-1t
BN BN.GK-1d
T2
T1
R2 Q2
R²and Q²
Correlation coefficient between original and permutted BN and BN.GK-1d
BN BN.GK-1v
T2
T1
R2 Q2
R²and Q²
Correlation coefficient between original and permutted BN and BN.GK-1v
BN BN.GK-1cns
T2
T1
R2 Q2
R²and Q²
Correlation coefficient between original and permutted BN and BN.GK-1cns
BN BN.GK-1o
T2
T1
R2 Q2
R²and Q²
Correlation coefficient between original and permutted BN and BN.GK-1o

Fig. S3. Loadings plots from O-PLS models performed on adipose tissue extracts from each congenic BN.GK strain against the BN control

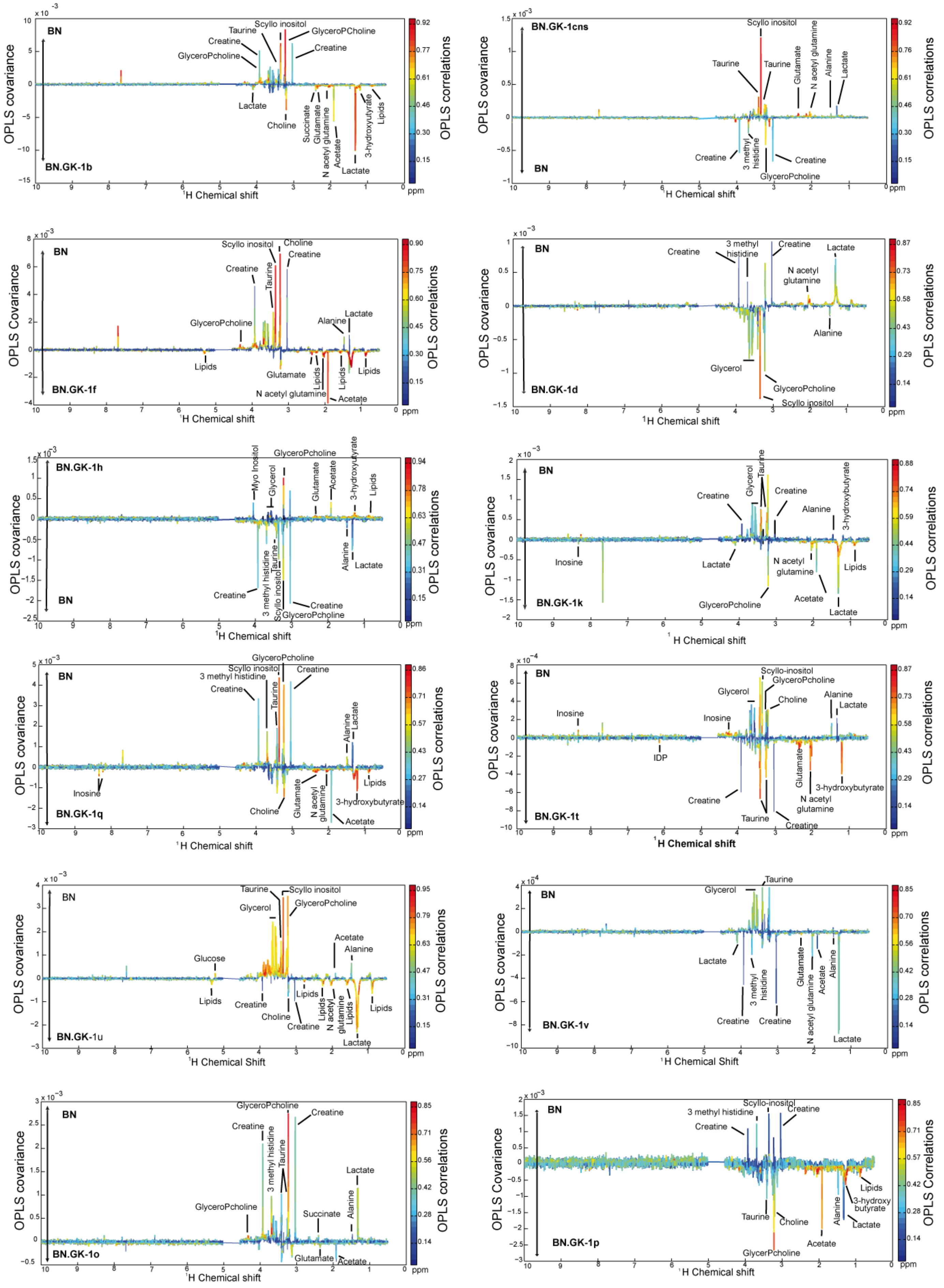

Fig. S4. Score plot from O-PLS model of each region of chromsome 1 against other regions and corresponding cross-validation with 200 permutations.

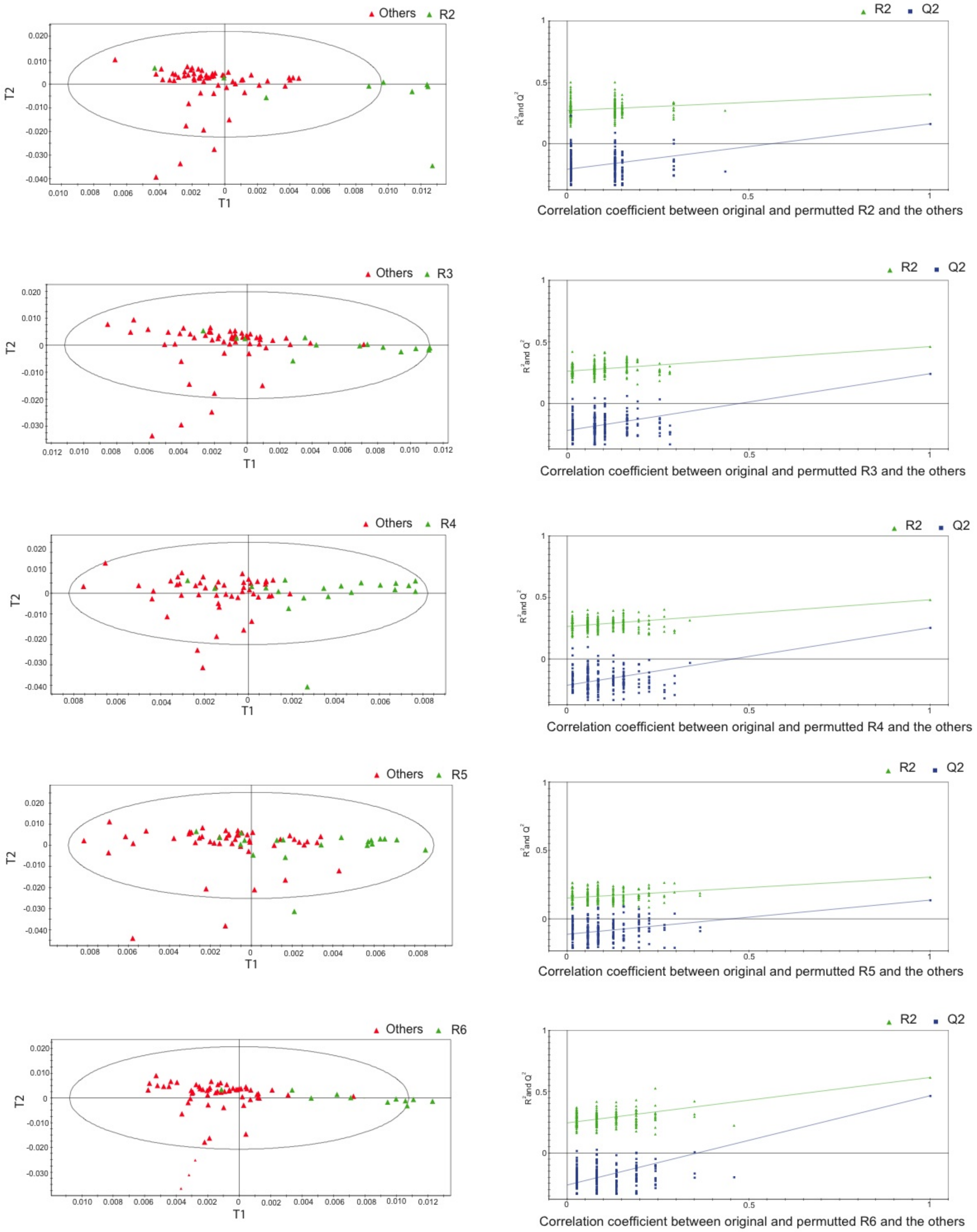

Fig. S4 (Cntd)

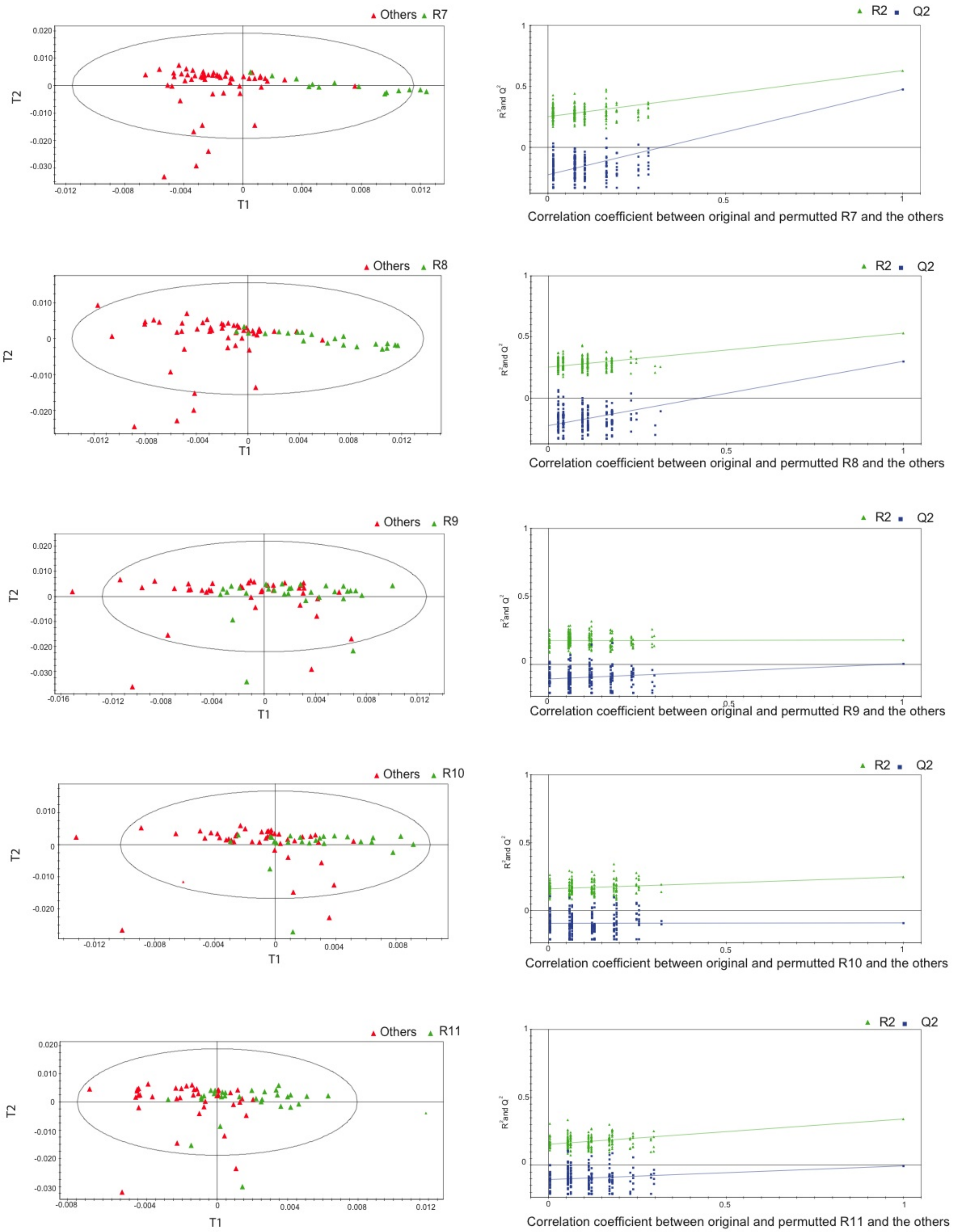
Others R7
T2
T1
R2 Q2
R^2 and Q^2
Correlation coefficient between original and permutted R7 and the others
Others R8
T2
T1
R2 Q2
R^2 and Q^2
Correlation coefficient between original and permutted R8 and the others
Others R9
T2
T1
R2 Q2
R^2 and Q^2
Correlation coefficient between original and permutted R9 and the others
Others R10
T2
T1
R2 Q2
R^2 and Q^2
Correlation coefficient between original and permutted R10 and the others
Others R11
T2
T1
R2 Q2
R^2 and Q^2
Correlation coefficient between original and permutted R11 and the others

Fig . S5. Loadings plots from O-PLS models performed for each region of chromosome 1 that can be dissected in the BN.GK congenic series against the other regions.

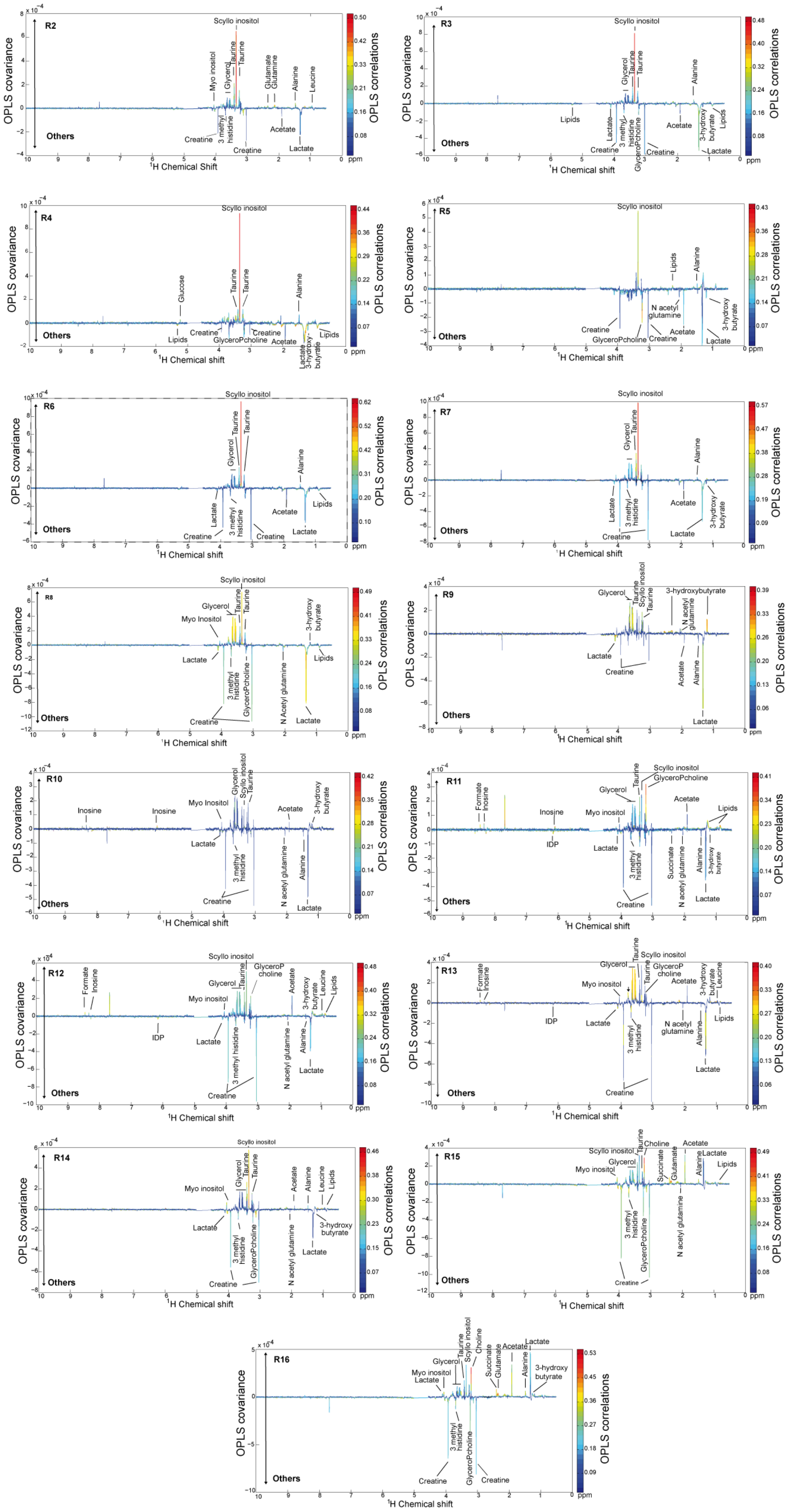

Supplement: Additional file 2: — Supplementary Figures S1–S5. (PDF 2442 kb) [file 13073_2016_352_MOESM2_ESM.pdf]
